# Influence of Prenatal Drug Exposure, Maternal Inflammation, and Parental Aging on the Development of Autism Spectrum Disorder

**DOI:** 10.3389/fpsyt.2022.821455

**Published:** 2022-02-09

**Authors:** Atsushi Sato, Hiroko Kotajima-Murakami, Miho Tanaka, Yoshihisa Katoh, Kazutaka Ikeda

**Affiliations:** ^1^Department of Pediatrics, The University of Tokyo Hospital, Tokyo, Japan; ^2^Addictive Substance Project, Tokyo Metropolitan Institute of Medical Science, Tokyo, Japan; ^3^Department of Psychiatry, Graduate School of Medicine, The University of Tokyo, Tokyo, Japan; ^4^Department of Obstetrics and Gynecology, Graduate School of Medicine, The University of Tokyo, Tokyo, Japan

**Keywords:** autism spectrum disorder, prenatal drug exposure, valproic acid, selective serotonin reuptake inhibitor, maternal immune activation, hypertensive disorders of pregnancy, advanced parental age

## Abstract

Autism spectrum disorder (ASD) affects reciprocal social interaction and produces abnormal repetitive, restrictive behaviors and interests. The diverse causes of ASD are divided into genetic alterations and environmental risks. The prevalence of ASD has been rising for several decades, which might be related to environmental risks as it is difficult to consider that the prevalence of genetic disorders related to ASD would increase suddenly. The latter includes (1) exposure to medications, such as valproic acid (VPA) and selective serotonin reuptake inhibitors (SSRIs) (2), maternal complications during pregnancy, including infection and hypertensive disorders of pregnancy, and (3) high parental age. Epidemiological studies have indicated a pathogenetic role of prenatal exposure to VPA and maternal inflammation in the development of ASD. VPA is considered to exert its deleterious effects on the fetal brain through several distinct mechanisms, such as alterations of γ-aminobutyric acid signaling, the inhibition of histone deacetylase, the disruption of folic acid metabolism, and the activation of mammalian target of rapamycin. Maternal inflammation that is caused by different stimuli converges on a higher load of proinflammatory cytokines in the fetal brain. Rodent models of maternal exposure to SSRIs generate ASD-like behavior in offspring, but clinical correlations with these preclinical findings are inconclusive. Hypertensive disorders of pregnancy and advanced parental age increase the risk of ASD in humans, but the mechanisms have been poorly investigated in animal models. Evidence of the mechanisms by which environmental factors are related to ASD is discussed, which may contribute to the development of preventive and therapeutic interventions for ASD.

## Introduction

Autism spectrum disorder (ASD) is a neurodevelopmental disorder with two symptomatic domains: impairments in reciprocal social interaction and repetitive and restrictive behaviors and interests (1). The prevalence of ASD is estimated to be 0.76% worldwide (2), varying across geographic regions, from 0.5% in Asia (3) to 2.5% in the United States (4). Compared with other psychiatric disorders, such as attention-deficit/hyperactivity disorder, ASD has many unanswered questions. There is no evidence of ASD remission in adulthood (2), and there are no effective pharmacotherapies for core symptoms of ASD. Understanding the pathomechanisms of ASD is necessary to develop preventive and therapeutic interventions.

The causes of ASD are divided into genetic alterations and environmental risk factors. Early studies of monozygotic and dizygotic twins estimated that the heritability of ASD is as high as 90% (5). However, subsequent research with improved methodologies showed heritability of 50–60%. Most of this heritability was attributed to common genetic variations, whereas the contribution of rare inherited mutations is lower (6). At the time of this writing, more than 1,200 genes and 2,200 copy number variations are listed in the Autism Database (7) as implicated in ASD (8).

Some of these genes cause congenital disorders, such as Fragile X syndrome and tuberous sclerosis complex. Both of these disorders have been extensively studied in both humans and animal models (9). Genetic analyses of familial ASD cases with no other specific manifestation have also unveiled many copy number variations and genetic mutations (10–12). The introduction of these genetic alterations in animals has contributed to our understanding of common neuronal mechanisms of ASD. For example, brains from mouse models of tuberous sclerosis complex (13, 14) and PTEN tumor hamartoma syndrome (15) exhibited the hyperactivity of mammalian target of rapamycin (mTOR) complex 1 (mTORC1). The inhibition of mTORC1 by rapamycin reversed ASD-related behavioral abnormalities (13–15). Mutations of *SHANK2, SHANK3, NLGN4*, and *NRXN1*α are considered to cause ASD by altering synaptic connectivity and neuronal excitability, resulting in excitatory/inhibitory (E/I) imbalance (16). A recent large-scale exome sequencing study found significant mutations of 102 genes, most of which were enriched in the excitatory and inhibitory neuronal lineage (17). Investigation of these genetic models has revealed the neuropathological findings in ASD (16). The brain of neuron-specific *Pten* knockout mice exhibited hypertrophy of hippocampal neurons (15). Synaptic density was increased in the cerebellum of *Tsc1*-deficient Purkinje mice (13) and stem cells carrying ASD-related *NLGN4* mutations (16). Lower neuronal excitability was found in *Tsc1*-deficient Purkinje cells (13) and *SHANK3*-deficient neurons (16), whereas neuronal excitability was enhanced in *CNTN*5^+/−^ and *EHMT*2^+/−^ cells (16).

The aforementioned evidence, however, does not necessarily explain the increasing prevalence of ASD over recent decades (i.e., from 0.4/1,000 to 2/1,000 in the 1970s to 1.5% or higher in recent studies) (18–20). According to recent studies, environmental risk factors account for the remaining 40–50% of ASD cases (6, 21), which might partially explain the increase in ASD prevalence. Meta-analyses have indicated several significant risk factors, such as advanced parental age, prenatal exposure to antidepressants, and complications during pregnancy (21–23). In contrast to epidemiological evidence, some of these risks are difficult to be reproduced without causing deleterious alterations in rodents, such as premature birth (24, 25) and low birth weight (26, 27), which hampers our understanding of whether and how these perinatal problems might contribute to ASD. The environment-induced ASD models discussed in this review also exhibit neuropathological features similar to those found in genetic models of ASD. However, the relation between each environmental risk and specific feature appears to be complicated. For example, prenatal exposure to valproic acid (VPA) results in ASD through different mechanisms, including disrupted γ-aminobutyric acid (GABA) signaling and mTORC1 hyperactivity. Shorter dendrites and reduced synapse pruning are observed in different ASD models, such as prenatal exposure to selective serotonin transporter inhibitors (SSRIs) and maternal inflammation.

**Figure 1:**
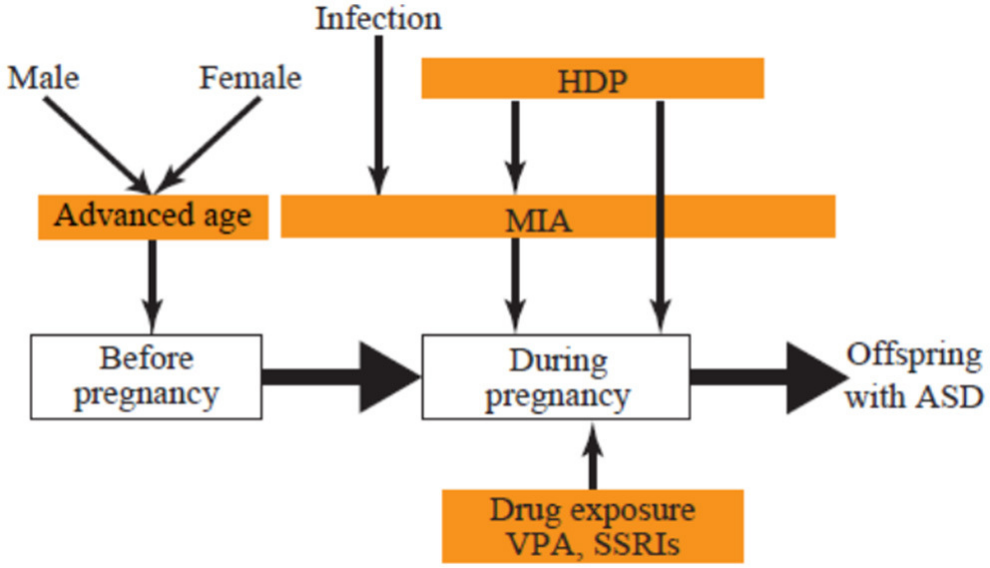
Developmental stage and environmental ASD risks. ASD, autism spectrum disorder; HDP, hypertensive disorders of pregnancy; MIA, maternal immune activation; SSRI, selective serotonin reuptake inhibitor; VPA, valproic acid.

The present review focuses on three groups of environmental risks of ASD: prenatal exposure to drugs, maternal complications during pregnancy, and advanced parental age. We discuss how each of these leads to ASD to better understand the mechanisms, with the goal of mitigating risk and developing potential therapeutics (Figure 1).

## Prenatal Exposure to Drugs

Exposure to medications during pregnancy is a well-known risk factor for ASD (21–23). Considering the abundance of research on mechanisms, VPA and SSRIs are discussed in this review.

### VPA

VPA has been clinically used as an antiepileptic drug for more than 50 years, and it is listed as a first-line drug for generalized epilepsy (28). Other indications of VPA include the prevention of migraine (29) and treatment of acute mania (30). It has a simple molecular structure (2-propylepntanoic acid) and appears to exert its clinical effects through various mechanisms, including the inhibition of GABA transaminase, voltage-gated Na^+^ channels, and T-type Ca^2+^ channels (29).

Despite its excellent clinical efficacy, congenital malformations and neurodevelopmental problems have been observed in children of mothers who took VPA during pregnancy (31). Congenital malformations that are related to VPA include neural tube defects, congenital heart disease, and cleft palate (32). Prenatal exposure to VPA increases the risk of ASD, but other antiepileptic drugs, such as carbamazepine and lamotrigine, do not (33). Children of mothers who used high-dose VPA (>800 mg/day) during pregnancy had a lower IQ at 6 years of age. Low-dose VPA (800 mg/day or less) did not affect IQ in children but was associated with impairments in verbal ability compared with other antiepileptic drugs (34). Prenatal exposure to carbamazepine or clonazepam did not affect the prevalence of ASD in offspring (33), implying that the inhibition of Na^+^ channels or potentiation of GABAergic signaling (i.e., effects of VPA) is insufficient to cause ASD. As discussed below, other pharmacological effects of VPA, either alone or combined, may be required. Based on these data, women who are considering becoming pregnant are now recommended to avoid using high-dose VPA by switching to other antiepileptic drugs or reducing VPA to dose to 600 mg/day or less before pregnancy (35). By contrast, those who are found to be pregnant while taking VPA are not advised to switch from VPA to other medications in order to avoid the risk of worsening epilepsy.

In accordance with the human data, rodents that were exposed to VPA *in utero* exhibited similar congenital anomalies and cognitive deficits (36). Rodent models of VPA-induced ASD are generated by administering higher doses of VPA after conception (see Table 1), whereas human mothers have an increased risk of having offspring with ASD when they become pregnant while taking VPA (>800 mg/day ≈15 mg/kg/day for increasing the risk of ASD). The methodology by which VPA is used to induce ASD-like behavior is well established, which sheds light on several pathways that lead to ASD.

**Table 1:** Postnatal treatment in ASD rodent models of prenatal exposure to VPA.

**Species/strain**	**Dose/route/time of exposure**	**Outcome**	**Treatment/route/time of exposure**	**References**
ICR mice	300 mg/kg, s.c., E10	↓ Sociability ↓ Social preference, social interaction	0.5–1 mg/kg CP465022, i.p., 30 min before testing 0.15–0.3 mg/kg PF4778574, i.p., 30 min before testing	(37)
ICR mice	300 mg/kg, s.c., E10 400 mg/kg, s.c., E12	↓ Sociability, social preference ↑ Marble burying, self-grooming	0.3 mg/kg donepezil, i.p., P14–40	(38)
C57BL/6 mice	500 mg/kg, i.p., E11	↓ Sociability ↑ Marble burying	30 mg/kg BrBzGCp2, i.p., 10 h before testing	(39)
C57BL/6J mice	600 mg/kg, s.c., E12	↓ Sociability	5 mg/kg rapamycin, i.p., 5 days	(40)
Sprague-Dawley rats	400 mg/kg, s.c., E12	↓ Sociability, social preference ↑ Self-grooming	0.3 mg/kg MK-801, i.p., 30 min before testing 30 mg/kg memantine, i.p., 30 min before testing	(41)
Sprague-Dawley rats	400 mg/kg, s.c., E12	↓ Sociability, social preference ↑ Self-grooming	25–100 mg/kg agmatine, i.p., 30 min before testing	(42)
Sprague-Dawley rats	500 mg/kg, i.p., E12 or E13	↓ Sociability	DBS in bilateral CTN, applied for 3 days	(43)
C57BL/6J mice	500 mg/kg, i.p., E12.5	↓ Sociability, social preference ↑ Marble burying, self-grooming	10 nM clonazepam, i.c., in mPFC, 30 min before testing	(44)
C57BL/6J mice	500 mg/kg, i.p., E12.5	↓ Sociability ↑ Marble burying	10–15 mg/kg E100, i.p., P44–65 1 mg/kg donepezil, i.p., P44–65	(45)
ICR mice	500 mg/kg, i.p., E12.5	↓ Social interaction	50–200 μg/kg oxytocin, i.n., 14 days	(46)
ICR mice	500 mg/kg, i.p., E12.5	↓ Social interaction	0.2 mg/kg risperidone, i.p.,14 days 3 mg/kg aripiprazole, i.p., 14 days	(47)
ICR mice	500 mg/kg, i.p., E12.5	↓ Sociability, social interaction ↑ Marble burying	80–160 μmol/kg betaine, s.c., 20 h before testing	(48)
Tuck-Ordinary mice	500 mg/kg, i.p., E12.5	↓ Sociability, social preference ↑ Marble burying, stereotypy, sociability, social interaction, USVs, marble burying, head-dipping	10–15 mg/kg DL77, i.p., 21 days 1 mg/kg donepezil, i.p., 21 days	(49)
129 × C57BL/6J mice	600 mg/kg, i.p., E12.5	↓ Sociability, social preference, USVs ↑ Marble burying	Knock-in of *Oxtr* in bilateral LS	(50)
C57BL/6 mice	600 mg/kg, i.p., E12.5	↓ Sociability, spontaneous alternation	7.5 mg/kg KU55933, i.n., P40	(51)
C57BL/6 mice	600 mg/kg, s.c., E12.5	↓ Sociability ↑ Marble burying, self-grooming	10 mg/kg TC-2153, i.p., 3 h before testing	(52)
C57BL/6J mice	600 mg/kg, s.c., E12.5	↓ Social interaction	10 mg/kg rapamycin, i.p., 2 days	(53)
Sprague-Dawley rats	400 mg/kg, i.p., E12.5	↓ Sociability, social preference ↑ Self-grooming	1 mg/kg rapamycin, i.p., P23–33	(54)
Wistar rats	450 mg/kg, i.p., E12.5	↓ Sociability, social preference, USVs ↑ Self-grooming	3 μg arginine vasopressin, s.c., P1–7	(55)
Sprague-Dawley rats	500 mg/kg, i.p., E12.5	↓ Sociability, social interaction ↑ Marble burying	2.5 μg wortmannin, i.c., in bilateral LA, 30 min before testing	(56)
Sprague-Dawley rats	500 mg/kg, i.p., E12.5	↓ Sociability	DBS in right mPFC, applied for 7 days	(57)
Sprague-Dawley rats	500 mg/kg, i.p., E12.5	↓ Sociability, social preference, spontaneous alternation ↑ Self-grooming	50–500 mg/kg metformin, p.o., P21–50	(58)
Sprague-Dawley rats	500 mg/kg, i.p., E12.5	↓ Sociability ↑ Marble burying, self-grooming	Diet enriched with fenofibrate (~200 mg/kg), P21–120	(59)
Sprague-Dawley rats	500 mg/kg, i.p., E12.5	↓ Sociability, social preference ↑ Marble burying	0.05 mg/kg URB597, i.p., 2 h before testing	(60)
Wistar rats	500 mg/kg (route not specified), E12.5	↓ Sociability, social interaction, USVs ↑ Head-dipping	1–2.5 ml/kg URB597, i.p., 30 min or 2 h before testing	(61)
Wistar rats	500 mg/kg, i.p., E12.5	↓ Sociability, social interaction, USVs ↑ Marble burying, head-dipping	0.05 mg/kg URB597, i.p., 2 h before testing	(62)
Wistar rats	500 mg/kg, i.p., E12.5	↓ Sociability ↑ Marble burying	10 μg D-cycloserine, i.c., in bilateral LA, 30 min before testing	(63)
Wistar rats	500 mg/kg, i.p., E12.5	↓ Sociability, social interaction, spontaneous alternation	10–20 mg/kg pioglitazone, p.o., P21–48	(64)
Wistar rats	500 mg/kg, i.p., E12.5	↓ Sociability, social interaction, spontaneous alternation	100–200 mg/kg fenofibrate, p.o., P21–48	(65)
Wistar rats	500 mg/kg, i.p., E12.5	↓ Sociability, social preference ↑ Self-grooming	20–100 mg/kg cannabidivarin, i.p., P34–58	(66)
Wistar rats	500 mg/kg, i.p., E12.5	↓ Sociability, social preference	1 mg/kg LP-211, i.p., P21–27	(67)
Wistar rats	500 mg/kg, i.p., E12.5	↓ Sociability, social preference, spontaneous alternation	10–20 mg/kg vinpocetine, p.o., P21–48	(68)
Wistar rats	500 mg/kg, i.p., E12.5	↓ Sociability, social preference, spontaneous alternation	3–30 mg/kg papaverine, i.p., P21–48	(69)
Wistar rats	500 mg/kg, i.p., E12.5	↓ Sociability, social preference, spontaneous alternation	30–60 mg/kg cilostazol, p.o., P21–48	(70)
Sprague-Dawley rats	600 mg/kg, s.c., E12.5	↓ Sociability	10 mg/kg PF3845, i.p., 2 h before testing	(71)
Sprague-Dawley rats	600 mg/kg, i.p., E12.5	↓ Social interaction	2.5 ml/kg cerebrolysin (route not specified),14 days	(72)
Sprague-Dawley rats	600 mg/kg, p.o., E12.5	↓ Sociability, social preference, social interaction, spontaneous alternation ↑ Self-grooming	1–30 mg/kg 5-ALA, p.o., P21–56 12 μg/kg oxytocin, i.n., P21–56	(73)
Sprague-Dawley rats	600 mg/kg, i.p., E12.5	↓ Sociability	3.5 mg/kg MS-275, i.p., P35–42 6 mg/kg retinoic acid, p.o., P35–42	(74)
Wistar rats	600 mg/kg, i.p., E12.5	↓ Sociability, social interaction ↑ Stereotypy	Environmental enrichment, P22–35	(75)
Wistar rats	600 mg/kg, i.p., E12.5	↓ Olfactory habituation/dishabituation, social interaction ↑ Self-grooming	80,000 IU/kg vitamin D3, i.m., P12	(76)
Wistar rats	600 mg/kg, i.p., E12.5	↓ Sociability, social preference	1 mg/kg fingolimod, p.o., P15–35	(77)
Wistar rats	600 mg/kg, i.p., E12.5	↓ Social interaction ↑Self-grooming	4 mg/kg rapamycin, p.o., P24–34	(78)
Wistar rats	600 mg/kg, i.p., E12.5	↓ Sociability ↑ Self-grooming	20 μg oxytocin, i.n., P40 3 μg oxytocin, s.c., P0–6	(79)
Wistar rats	600 mg/kg, i.p., E12.5	↓ Sociability, social interaction ↑ Self-grooming	10 mg/kg Dapt, i.p., 10 days	(80)
Wistar rats	600 mg/kg, s.c., E12.5	↓ Sociability, social preference ↑ Self-grooming	0.03 mg/kg MK-801, i.p., P6–10	(81)
Wistar rats	600 mg/kg, i.p., E12.5	↓ Olfactory habituation/dishabituation, social interaction	Diet enriched with n-6 polyunsaturated fatty acid, P21–**77**	(82)
Wistar rats	600 mg/kg, i.p., E12.5	↓ Sociability, social preference, social recognition memory	0.03–0.1 mg/kg cariprazine, p.o., 7 days 0.1 mg/kg risperidone, p.o., 7 days 1 mg/kg aripiprazole, p.o., 7 days	(83)
Wistar rats	600 mg/kg, i.p., E12.5	↓ Sociability, social preference ↑ Marble burying	30 mg/kg dextromethorphan, i.p., P23–43	(84)
Wistar rats	600 mg/kg, i.p., E12.5	↓ Sociability, social preference ↑ Marble burying, self-grooming	1–10 mg/kg JZL184, i.p., PND21–34 40 mg/kg JZL184, i.p., 2 h before testing	(85)
C57BL/6Hsd mice	600 mg/kg, s.c., E13	↓ USVs, sociability ↑ Self-grooming	10 mg/kg MPEP, i.p., 5 min before testing	(81)
C57BL/6Hsd mice	600 mg/kg, s.c., E13	↑ Marble burying, self-grooming	20 mg/kg MPEP, i.p., 10 min before testing	(86)
C57BL/6J mice	400 mg/kg, i.p., E13.5	↓ Social interaction, spontaneous alternation ↑ Rearing, self-grooming	200 mg/kg sodium phenylbutyrate, i.p., P21–63 200 mg/kg sodium phenylbutyrate, i.p., 30 min before testing 320 mg/kg D-cycloserine, i.p., P21–63	(87)
C57BL/6J mice DBA/2 mice	600 mg/kg, i.p., E13.5	↓ Social preference	30 mg/kg resveratrol, i.p., 24 h before testing	(88)
Sprague-Dawley rats	(Not found in text)	↓ Social interaction	0.63–10 mg/kg F17464, i.p., 30 min before testing 0.16–5 mg/kg fenobam, i.p., 30 min before testing 0.16–2.5 mg/kg memantine, i.p., 30 min before testing	(89)

*BrBzGCp2, S-p-bromobenzylglutathione cyclopentyl diester; CTN, central thalamic nuclei; Dapt, (3,5-difluorophenacetyl)-L-alanyl-S-phenylglycine-2-butyl Ester; DBS, deep brain stimulation; E, embryonic day; i.c., intracerebral; i.m., intramuscular; i.n., intranasal; i.p., intraperitoneal; LA, lateral amygdala; LS, lateral septum; mPFC, medial prefrontal cortex; P, postnatal day; p.o., per os; s.c., subcutaneous; USVs, ultrasound vocalizations*.

#### Alterations of GABAergic Signaling

Although it is one of the main mechanisms of action by which clinical effects manifest, remaining unclear is whether the inhibition of GABA transaminase by VPA is critical for disturbing the development of fetal brains. GABA is synthesized by glutamic acid decarboxylase (GAD), which converts glutamate to GABA, and degraded by GABA transaminase. GABA exerts its action by binding to specific receptors, GABA_A_ or GABA_B_ receptors, which results in the opening of Cl^−^ channels. GABA stimulates the influx of Cl^−^ ions in the mature brain, resulting in a decrease in intracellular transmembrane potential (90). However, neurons in the immature brain have higher concentrations of intracellular Cl^−^ because of higher activity of Na-K-Cl cotransporter 1 (NKCC1) that passes Cl^−^ into neurons. The binding of GABA to GABA receptors in immature neurons causes the efflux of Cl^−^ and depolarization of neurons (91). This excitatory signaling via GABA is important for normal development of the immature brain, including neurogenesis and synapse formation (92, 93).

The mechanism by which VPA potentiates GABAergic signaling in the fetal brain may be related to the development of ASD. Prenatal treatment with a single dose of VPA, mainly on embryonic day (E) 12.5 in mice (44) and rats (94, 95) but as late as E17 in mice (96) reduced GAD expression in different brain regions that suggests a lower number of GABAergic neurons (94–96), and reduced GABA concentrations and the number of GABA receptors (44). These changes have been associated with impairments in fear conditioning, object recognition (94), and ASD-like social deficits (94, 95). Interestingly, the prenatal inhibition of GABAergic signaling with GABA receptor antagonists, such as picrotoxin administration on E10-12, suppressed neurogenesis in the fetal brain (97) and resulted in ASD-like social deficits in offspring (97, 98). These findings suggest that GABA signaling should be controlled within a limited range to maintain normal development of the fetal brain.

Another mechanism may involve the excitatory-to-inhibitory shift of GABAergic signaling during delivery. This shift may be triggered by oxytocin. Oxytocin blockade on the day before delivery resulted in persistent excitatory GABAergic signaling in the postnatal brain (99). Pups that were born to rats that received VPA on E12 also exhibited diminished excitatory-to-inhibitory shift of GABAergic signaling on postnatal day (P) 15. Maternal pretreatment with bumetanide, an inhibitor of NKCC1, restored the inhibitory GABAergic signaling, and pups of bumetanide-treated mothers exhibited the normalization of isolation-induced ultrasound vocalizations (USVs) (99). In an immature neuron model that utilized cultured cortical neurons that were prepared from P1 rats, 3-day VPA treatment immediately after preparation of the neurons reduced vesicular GABA transporter (VGAT) expression and the number of GABAergic synapses, which persisted for 10 days after cessation of VPA exposure (100). However, VPA did not reduce VGAT expression when treatment began after cultivating the neurons for 8 days. Considering that P1 in rats corresponds to approximately 23 weeks gestation in humans, and P8 around delivery (101), these findings suggest that mid-gestational exposure to VPA produces excitatory-dominant E/I imbalance by blocking the excitatory-to-inhibitory shift of GABAergic signaling or reducing VGAT expression and decreasing GABA synthesis in the postnatal brain. This is further supported by the therapeutic effect of the postnatal restoration of E/I balance for ASD. Postnatal intracerebral administration of the GABA_A_ receptor agonist clonazepam rescued deficient social novelty in mice that were exposed to VPA on E12.5, whereas the GABA_B_ receptor agonist baclofen did not (44). The postnatal suppression of glutamatergic signaling using the *N*-methyl-D-aspartate receptor antagonist MK-801 or metabotropic glutamate receptor 5 antagonist MPEP was similarly effective in restoring GABAergic signaling in rodents that were exposed to VPA on E12-13 (41, 81, 86, 102). Prenatal exposure to VPA may cause persistent excitatory-dominant E/I imbalance and ASD, which might be reversed by the postnatal correction of E/I balance.

#### Disturbances in Folic Acid Metabolism

The second mechanism by which VPA is implicated in ASD is the association between VPA and folic acid deficiency. Neural tube defects (i.e., a congenital malformation of the spinal cord in which the neural tube fails to close during embryogenesis and results in exposure of the “unclosed” spinal cord, also called spina bifida) were shown to be related to vitamin deficiencies, including folic acid deficiency (103). A randomized control study found that daily folic acid supplementation reduced the risk of neural tube defects, but other vitamins did not (104). Unexpectedly, children who were exposed to VPA *in utero* had a higher risk of neural tube defects (32) and ASD (33), similar to children who were born to women with folic acid deficiency during pregnancy. These observations raised the possibility that folic acid supplementation may be useful for the prevention of VPA-related conditions, such as neural tube defects and ASD. Folic acid supplementation before and during pregnancy significantly reduced the risk of ASD in children who were exposed to antiepileptic drugs, including VPA (105). Interestingly, folic acid supplementation lowered the risk of ASD in offspring to less than half, regardless of the concomitant use of antiepileptic drugs (106).

Despite these clinical findings that strongly suggest a similarity between prenatal exposure to VPA and folic acid deficiency in the development of ASD, little is known about the underlying mechanisms by which these two conditions result in ASD or the mechanisms by which folic acid prevents ASD. In a study of human placentas from women without epilepsy, VPA perfusion for 3 h reduced placental concentration of folic acid by ~30% and suppressed mRNA levels of the *FOLR1* gene, which encodes folate receptor α (107). VPA is also a non-competitive inhibitor of high-affinity folate receptors, such as folate receptor α (108), and may disturb folic acid metabolism (109). The preventive effect of maternal folic acid supplementation was recapitulated in rodents with regard to neural tube defects. VPA administration in pregnant ICR mice on E8 caused neural tube defects, which were prevented by oral folic acid administration prior to the VPA injection (110). A recent study investigated the effect of folic acid supplementation on VPA-induced ASD. Neuropathological changes in offspring that were prenatally exposed to VPA on E12.5 included an increase in dendritic spine density, an increase in the expression of vesicular glutamate transporter 1 (VGLUT1) and postsynaptic density 95 (PSD95; i.e., markers of excitatory neurons), and a decrease in the expression of GAD65 and gephyrin (i.e., markers of inhibitory neurons) (111), suggesting a net result of excitatory-dominant E/I imbalance. The offspring also exhibited ASD-like behavioral deficits that were dose-dependently prevented by maternal folic acid supplementation from E1 to E12.5 (111), which was consistent with the lower prevalence of ASD that correlates with maternal folic acid supplementation (33). Further studies should investigate how folic acid normalizes VPA-induced alterations in the fetal brain.

#### Inhibition of Histone Deacetylase

Two other mechanisms may also link *in utero* VPA exposure to ASD in offspring, although they seem less relevant to clinical effects: inhibition of histone deacetylase (HDAC) and activation of the mTORC1 signaling pathway. Trichostatin A, an established HDAC inhibitor, was teratogenic in *Xenopus* embryos similarly to VPA (112). VPA and trichostatin A also reduced VGAT expression in cortical neurons obtained from P1 rats (100). This change is considered to result in lower extracellular GABA levels and the disruption of E/I balance toward predominately excitatory transmission, which was observed in another study that employed a rat model of prenatal VPA exposure (111). Moreover, maternal exposure to VPA and trichostatin A on E12.5 decreased USVs and sociability (113). These alterations that are associated with VPA and trichostatin A appear to be caused by HDAC inhibition. Valpromide, an analog of VPA that lacks HDAC inhibitor activity, failed to recapitulate phenotypic changes that were induced by VPA. At the histological level, valpromide administration did not reduce VGAT expression in cortical neurons (100) or global gene expression in the embryonic telencephalon (114). The number of Nissl-positive cells was comparable in the prefrontal and somatosensory cortices when valpromide was administered (115). No congenital malformation (112), deficits in social interaction, anxiety-related behavior, or learning and memory deficits (115) were observed in rodent offspring that were exposed to valpromide *in utero*. These findings emphasize that the HDAC inhibition by VPA affects normal embryogenesis and neuronal development that leads to ASD.

#### Activation of mTORC1

Another mechanism of VPA-induced ASD is activation of the phosphoinositide 3-kinase (PI3K)/protein kinase B (Akt)/mTORC1 pathway (116). Briefly, the mTORC1 signaling pathway is stimulated by growth factors and altered energy levels, and activates mTORC1 that results in stimulation of cell growth and proliferation (117). These effects are mediated by the phosphorylation of downstream signaling molecules, including ribosomal protein S6 kinases (S6Ks) that regulate global protein synthesis, ULK-51-like kinase 1 (ULK1) that is involved in initiating macroautophagy, and eukaryotic translation initiation factor-4E (eIF4E)-binding proteins (4E-BPs) that control cap-dependent translation (117). mTORC1 hyperactivity underlies ASD in human diseases that are caused by mutations of genes that are upstream of mTORC1, such as tuberous sclerosis complex (13, 14) and PTEN tumor hamartoma syndrome (15). Rodent models in which genes that are downstream of mTORC1 were deleted also exhibited ASD-like behavioral changes that were associated with excessive protein synthesis, impairments in autophagy, and alterations of the gene translation profile (118). In summary, alterations of cell growth and proliferation that are under the control of mTORC1 result in impairments in social behaviors that are relevant to ASD.

Both mice (40, 53) and rats (54) that were exposed to VPA on E12 exhibited an increase in mTORC1 activity in the brain and ASD-like behaviors, such as a decrease in social interaction and increase in self-grooming (40, 53, 54). With regard to developmental delays in children who are prenatally exposed to VPA (34), VPA-exposed mice exhibited deficits in early postnatal development, including eye opening, the righting reflex, and performance in the hanging wire test (53). Related to these behavioral changes, postnatal brains of these animals exhibited signs of the suppression of autophagy (40, 54), one of the main consequences of mTORC1 hyperactivity (117, 118). The expression of a set of genes was also changed, including Fyb, which functions downstream of the mTORC1 signaling pathway (53). Deficits in behavior, autophagy, and gene expression were all reversed by postnatal treatment with the mTORC1 inhibitor rapamycin (40, 53, 54). The constitutive activation of mTORC1 is likely a treatable mechanism by which social behavior is disrupted in VPA-induced ASD.

### SSRIs

#### SSRIs, Serotonin Metabolism, and ASD in Humans

Serotonin (5-hydroxytryptamine [5-HT]) is a neurotransmitter that is involved in diverse brain functions, including motor activity and emotion. When released from presynaptic vesicles, 5-HT binds to post- and presynaptic receptors. Serotonin is then either degraded or collected by the serotonin transporter (SERT). Serotonin plays a critical role in neurodevelopment, including the development of serotonergic fibers (119). SSRIs are a group of drugs that inhibit the SERT and elevate extracellular 5-HT levels in the brain (120). SSRIs are used as a first-line treatment of major depressive disorder (121). The prevalence of depression during pregnancy is estimated to be as high as 10%, and its treatment is often indicated during pregnancy (122, 123).

The role of alterations of serotoninergic signaling in ASD was suggested by the observation that approximately 30% of individuals with ASD have high mean blood levels of 5-HT (124). The maternal use of SSRIs is speculated to elevate maternal 5-HT levels, which might affect fetal brain development and result in a higher risk of ASD in offspring. Case-control studies found a 2-fold increase in the risk of ASD in children of mothers who reported antidepressant use during pregnancy (125, 126). This effect was particularly strong when antidepressants were used during the first trimester of pregnancy, regardless of whether or not the mothers had depression (125). In another cohort study, the use of SSRIs during the second and third trimesters of pregnancy doubled the risk of ASD in offspring, regardless of a maternal history of depression (127). However, later studies reported inconsistent findings with regard to the association between antidepressant use during pregnancy and ASD in offspring (128). A meta-analysis reported an association between the pre-pregnancy maternal use of antidepressants and ASD in offspring and not during pregnancy (23). Another case-control study found that the risk of neurodevelopmental disorders, including ASD, was associated with maternal psychiatric conditions but not the maternal use of SSRIs during pregnancy (129). A nationwide cohort study in Finland reported the influence of prenatal SSRI use on a higher incidence of depression but not ASD (130). In summary, the relationship between ASD and maternal conditions, such as psychiatric comorbidity and antidepressant use, remains elusive and requires further epidemiological studies.

#### SSRIs, Serotonin Metabolism, and ASD in Animal Models

Investigations of pregnant rodents have improved our understanding of 5-HT dynamics and its alterations by SSRIs during pregnancy. Analyses of *Pet1* knockout mice, in which most dorsal raphe neurons lacked 5-HT, revealed that 5-HT in the fetal forebrain was of placental and not maternal or fetal origin (131). The blockade of SERTs using the serotonin-norepinephrine transporter inhibitor venlafaxine by gavage from E8 to E20 decreased placental weight and SERT expression in the placenta in rats (132). The inhibition of 5-HT signaling with the 5-HT_2_ receptor antagonist ketanserin by gavage from E15 to E20 reduced placental weight and placental blood flow in rats (133). SSRI use during pregnancy could result in lifelong consequences on the brain in offspring (134), but the underlying mechanism is complex, including fetal exposure to SSRIs and alterations of 5-HT supply from the placenta. Decreases in placental weight and blood flow that are associated with maternal treatment with SSRIs could also lower the supply of oxygen and nutrients to the fetus and result in lower offspring weight, which might affect fetal brain development.

In contrast to VPA, few studies have investigated how prenatal exposure to SSRIs affects social behavior in offspring (Table 2). At the behavioral level, fluoxetine administration in mice that began before pregnancy or from early gestation to late-gestation or delivery produced ASD-like behavioral deficits in offspring, decreased USVs in pups, disrupted social interaction, enhanced social dominance, and increased tactile hypersensitivity (135–137). Citalopram, an SSRI with particularly high specificity for blocking SERT compared with dopamine and norepinephrine transporters, also altered behavior in mouse offspring, decreased sociability, decreased social preference, decreased locomotor activity, and increased anxiety-related behavior when given during late gestation (138). In fetal brains that were exposed to fluoxetine, neurons in the prefrontal cortex exhibited a reduction of the frequency of inhibitory synaptic currents, and interneurons exhibited an increase in intrinsic and serotonin-induced excitability (136). Prefrontal cortex tissue from the fluoxetine-exposed brain exhibited high mRNA levels of 5-HT_2A_ receptor (136). Striatal extracts from mice that were prenatally exposed to citalopram expressed higher levels of NMDAR1 and CaMKIIα, which were associated with morphological changes in the striatal neurons and decreases in dendritic length, number, and branch patterns (138). Prenatal exposure to SSRIs is suggested to result in excessive 5-HT signaling and altered E/I balance.

**Table 2:** Rodent models of prenatal exposure to SSRIs.

**Species/strain**	**Compound/dose/route/time of exposure**	**Behavioral outcome**	**Treatment/route/time of exposure**	**References**
C57BL/6J mice	16 mg/kg fluoxetine, p.o., before mating to E16, delivery, or P14	↓ USVs, sociability	—	(135)
C57BL/6 mice	15 mg/kg fluoxetine, p.o., before mating to P14	↓ Sociability, social preference	—	(137)
C57BL/6J mice	0.6 mg/kg fluoxetine, i.p., E4–19	↓ Spontaneous alteration, social preference	0.01 mg/kg MDL100907, i.p., 30 min before testing	(136)
C57BL/6 mice	20 mg/kg citalopram, i.p., E13 to delivery	↓ Sociability, social preference	10 mg/kg memantine, i.p., 20 min before testing	(138)

*E, embryonic day; i.p., intraperitoneal; P, postnatal day; p.o., per os; USVs, ultrasound vocalizations*.

The effects of therapeutic interventions in these models also appear to be consistent with these findings. High 5-HT level in the brain that are caused by reexposure to fluoxetine in adulthood recovered tactile hypersensitivity (135). The 5-HT_2A_ receptor antagonist MDL100907 suppressed abnormal excitability in neurons in the prefrontal cortex and reversed social preference in a model of fluoxetine-induced ASD (136). In a citalopram model, high levels of NMDAR1 and CaMKIIα were normalized by postnatal treatment with memantine, which was associated with the recovery of sociability and social preference (138). More research is required to determine whether and how exposure to SSRIs *in utero* affects fetal brain development and causes social deficits.

The aforementioned interventions can be partially replicated by manipulating genes that are involved in 5-HT neurotransmission because 5-HT levels are consistently changed in these models (i.e., either elevated or depleted). The most extensively investigated models are *SERT* knockout mice and rats (139). In these animals, extracellular 5-HT levels are several times higher, and consequently the density of 5-HT_1A_ and 5-HT_1B_ receptors is decreased (140–144). Diverse behavioral phenotypes are observed in *SERT* knockout rodents, such as an increase in anxiety and fear (139), that may affect social behavior in *SERT* knockout animals. Intact sociability was observed in *Sert* ^−/−^ and *Sert* ^+/−^ mice, reflected by the time spent sniffing a novel mouse or a novel object (145). The heterozygous loss of *Sert* aggravated deficient sociability in *Pten*^+/−^ mice, a genetic model of ASD that is associated with the activation of mTORC1 activity (146). Reciprocal social interaction in the resident-intruder paradigm was unaffected in *Sert* ^−/−^ and *Sert*^+/−^ rats (147). A recent study reported deficits in social interaction, sociability, and social novelty in *Sert* ^−/−^ and *Sert* ^+/−^ mice (148). This was associated with high levels of 5-HT in the brain in *Sert* ^−/−^ mice but not in *Sert* ^+/−^ mice. Impairments in social interaction were ameliorated by restricting the dietary intake of tryptophan (i.e., the precursor of 5-HT), which lowers 5-HT levels in the brain (148). Constitutively elevated 5-HT levels are thus considered to disrupt social behavior.

Reducing 5-HT concentrations in the brain, opposite to *SERT* deletion, may also give rise to ASD. One method to reduce 5-HT levels is to introduce a gain-of-function mutation of the *SERT* gene. *SERT* Ala56 mice that expressed an ASD-associated variant in humans exhibited elevations of 5-HT clearance in the brain and ASD-related social impairments and repetitive behavior, but forebrain 5-HT levels did not change in the mutants (149). This increase in 5-HT clearance was reversed by MW150, a p38α mitogen-activated protein kinase inhibitor, which also normalized social dominance in the tube test (150). Brain 5-HT levels can also be depleted by deleting the tryptophan hydroxylase 2 (*TPH2*) gene, which is essential for synthesizing 5-HT in the brain. *TPH2* knockout mice exhibited diverse ASD-related behaviors, including impairments in social interaction, an increase in marble burying, and deficient early developmental milestones (151). Female *TPH2* knockout mice exhibited high levels of aggression against co-housed mice and an increase in defensive behavior when paired with a knockout mouse (152). These studies suggest that prenatal increases and decreases in fetal 5-HT levels may result in the subsequent development of ASD.

## Maternal Immune Activation

Another possible environmental risk factor for ASD is maternal inflammation that is induced by infection during pregnancy. Associations between ASD and prenatal infection with specific pathogens, such as rubella and cytomegalovirus, have been repeatedly reported (153). Meta-analyses revealed a mild but significant increase in the risk of ASD in children of mothers who experienced infection during pregnancy, regardless of the pathogen (154, 155). One study estimated that maternal infection accounts for 12–17% of ASD cases (155). The risk of ASD appears to correlate with the severity of maternal infection, in which the risk was further elevated when mothers required hospitalization because of the infection (154). In another meta-analysis, maternal fever during pregnancy, regardless of whether it was caused by infection, increased the risk of neurodevelopmental disorders (156). The invasion of pathogens does not appear to be essential for the development of ASD in offspring; instead, the maternal inflammatory response itself seems sufficient.

Maternal immune activation (MIA) and cytokine production following infection are likely central mechanisms that link maternal infection and ASD in offspring (157). Briefly, MIA-induced ASD appears to begin with the selective activation of Th1 cells, consequently resulting in high interleukin-6 (IL-6) levels, but other mechanisms may also mediate the development of ASD. IL-6 activates retinoic acid receptor-related orphan nuclear receptor γt (RORγt) in naive CD4^+^ T cells, which stimulates its differentiation into Th17 cells, Activated Th17 cells produce cytokines, including IL-17A that may be critically involved in the development of ASD (158–160). Microglia are local macrophages in the brain that mediate MIA and neurodevelopment. Normal functions of microglia include neurogenesis and synapse pruning, playing an important role in synaptic plasticity (161). The production of proinflammatory cytokines by MIA, such as IL-6 and IL-17, results in microglial activation, which suppresses synapse pruning particularly in the hippocampus and disrupts synapse function (162). The relation between MIA and the development of ASD in offspring has been demonstrated in both humans and in rodents.

### Maternal Infection, Immune Activation, and ASD in Humans

Findings are limited about markers that are suggestive that children with ASD were exposed to MIA *in utero*. The Early markers for Autism study found that high levels of IL-4, IL-5 and interferon-γ (IFN-γ) in maternal serum at 15–19 weeks of gestation were associated with a 50% higher risk of ASD, whereas high levels of IL-2, IL-4 and IL-6 was associated with developmental delay in the absence of ASD (163). Using a larger sample set, a subsequent analysis found high levels of many cytokines, including IL-1α and IL-6, in mothers of children with ASD and developmental delay compared with children with ASD without developmental delay and controls (164). The cytokine profiles of neonates that were exposed to MIA may reflect immunological alterations in their mothers, but the presence of neonatal cytokines was determined using dried blood spots from the neonates, and cytokines in these samples might be unstable. A study did not find changes in these cytokines, such as increase in IL-6; instead, Th1 and Th2 cytokine levels decreased (165) or were comparable between ASD children and controls (166). High IL-4 levels were associated with a higher risk of severe ASD (odds ratio o = 1.4), and higher IL-1β were associated with mild or moderate ASD (odds ratio = 3.02) (167). Children with ASD had high levels of IL-6 and IL-8 during neonatal periods compared with controls (168). No differences were found in cytokine levels between children with developmental delay in the absence of ASD and controls (167, 168).

High levels of proinflammatory cytokines may persist beyond the neonatal period. Two-to 5-year-old children with ASD had high plasma levels of the proinflammatory cytokine IL-6 compared with age-matched typically developing controls and those with developmental disabilities other than ASD (169). Brains of ASD patients also had high cytokine levels, including IL-6 and the Th1 cytokine IFN-γ, whereas levels of Th2 cytokines (i.e., IL-4, IL-5, and IL-10) were not elevated in brains in ASD patients (170). An increase in serum IL-17A levels was also detected in 6- to 11-year-old ASD children (171). The phytohemagglutinin-induced stimulation of peripheral blood mononuclear cells resulted in the production of IL-17 but not Th2 cytokines IL-4 and IL-13 in 2- to 5-year-old ASD children, whereas cytokine levels at baseline were comparable between ASD children and controls (172). Plasma levels of IL-17 and IL-1β remained elevated in 7- to 15-year-old ASD individuals (173). Heavy cytokine burden may be related to a more severe ASD phenotype. Children with the regressive form of ASD exhibited a significant increase in IL-1β and IL-6 levels, whereas those without the regressive form did not (169). Most children who had high serum IL-17A levels had severe ASD, based on the Childhood Autism Rating Scale (171). Another study, however, did not find a correlation between the cytokine profile and clinical variables, including the severity of ASD, was not found (173). Altogether, exposure to IL-6 and IL-17, rather than direct effects of pathogens, may be important for MIA-induced ASD.

### Modeling Maternal Immune Activation in Animals

To investigate the mechanisms of MIA-related ASD in disease models, two approaches have been utilized. One is to inoculate human pathogens, and the other is to administer proinflammatory compounds (Table 3).

**Table 3:** Maternal immune activation models of ASD in rodents.

**Species/strain**	**Compound/dose/route/time of exposure**	**Behavioral outcome**	**Treatment/route/time of exposure**	**References**
BALB/c mice	6,000 pfu influenza A, i.n., E9.5	↓ Social interaction	—	(174)
BALB/c mice	75–600 pfu Influenza A (H3N2), i.n., E9.5	↓ Social interaction	—	(175)
Balb/c mice	10^8^ cfu *Mycobacterium tuberculosis*, aerosol infection, E12.5	↓ Sociability, social preference ↑ Self-grooming	—	(176)
C57BL/6J mice	200 μg/kg Staphylococcal enterotoxin A or B, i.p., E12.5	↓ Sociability	—	(177)
C57BL/6 mice	90 μg soluble tachyzoite antigen from *Toxoplasma gondii*, i.p., E14.5	↓ Sociability, social preference, social interaction ↑ Marble burying, self-grooming	Transfer of maternal regulatory T cells	(178)
Lewis rats	10^8^-10^9^ cfu group B Streptococcus, i.p., E19	↓ Social interaction, USVs	—	(179)
Rats	10^9^ cfu group B Streptococcus, i.p., every 12 h from E19 to delivery	↓ Olfactory discrimination, social interaction	—	(180)
C57BL/6N mice	5 mg/kg poly(I:C), i.v., E9	↓ Sociability ↑ Marble burying	Maternal injection of 1,25OHD, s.c., E9	(181)
C57BL/6 mice	5 mg/kg poly(I:C), i.p., E10.5, E12.5, and E14.5	↓ Sociability ↑ Marble burying, USVs	—	(182)
C57BL/6 mice	20 mg/kg poly(I:C), i.p., E11.5–12.5	↓ Sociability, USVs ↑ Marble burying	Maternal injection of anti-IL-17A antibody, i.p., E11.5	(183)
C57BL/6J mice	3 mg/kg poly(I:C), i.p., E12.5	↓ Sociability	Genetic removal of *Nox1*	(184)
BTBR mice	20 mg/kg poly(I:C), i.p., E12.5	↓ Sociability ↑ Marble burying, self-grooming (M), USVs (PND8, 10)	—	(185)
C57BL/6J mice	20 mg/kg poly(I:C), i.p., E12.5	↓ Sociability	Maternal injection of anti-IL-6 antibody, i.p., E12.5	(186)
C57BL/6J mice	20 mg/kg poly(I:C), i.p., E12.5	↓ Sociability, reversal learning ↑ Self-grooming	—	(187)
C57BL/6J mice	20 mg/kg poly(I:C), i.p., E12.5	↓ Sociability	—	(188)
C57BL/6J mice	20 mg/kg poly(I:C), i.p., E12.5	↓ Sociability, USVs ↑ Marble burying	20 mg/kg RS102895, i.p., P10 Genetic removal of CCR2 in monocytes	(189)
C57BL/6J mice	20 mg/kg poly(I:C), i.p., E12.5	↓ Social preference	—	(190)
C57BL/6J mice	20 mg/kg poly(I:C), i.p., E12.5	↓ Sociability, social preference, social interaction ↑ Marble burying	0.0625 mg/kg clonazepam, i.p., single injection	(191)
C57BL/6N mice	20 mg/kg poly(I:C), i.p., E12.5	↑ Marble burying	Maternal diet enriched with choline	(192)
C57BL/6N mice	20 mg/kg poly(I:C), i.p., E12.5	↓ Sociability ↑ Marble burying	Genetic deletion of *Il6ra* in placental trophoblasts	(193)
C57BL/6 mice	20 mg/kg poly(I:C), i.p., E12.5	↓ Sociability ↑ Marble burying (M)	—	(194)
C57BL/6 mice	20 mg/kg poly(I:C), i.p., E12.5	↓ Sociability, USV duration ↑ Marble burying	Maternal injection of anti-IL-17A antibody, i.p., E12.5	(195)
C57BL/6 mice	20 mg/kg poly(I:C), i.p., E12.5	↓ Sociability, social preference ↑ Self-grooming, USVs	Maternal diet enriched with docosahexaenoic acid	(196)
C57BL/6 mice	20 mg/kg poly(I:C), i.p., E12.5	↓ Social preference ↑ Self-grooming	—	(197)
C57 mice	20 mg/kg poly(I:C), i.p., E12.5	↓ Sociability ↑ Marble burying, USVs (PND10)	—	(185)
FVB/N EGFP-Tg mice	20 mg/kg poly(I:C), i.p., E12.5	↓ Social recognition, USVs (PND6, 8) ↑ Marble burying, USVs (PND10)	—	(198)
C57BL/6J mice	50 mg/kg poly(I:C), s.c., E12.5	↓ Sociability, social preference	40 mg/kg resveratrol, s.c., E9.5–14.5	(199)
C57BL/6 mice	2 mg/kg poly(I:C), i.p., E12.5 3 mg/kg poly(I:C), i.p., E12.5 and 1.5 mg/kg poly(I:C), i.p., E17.5	↓ Sociability	10–20 mg/kg suramin, i.p. weekly beginning at 6 weeks	(200)
C57BL/6J mice	3 mg/kg poly(I:C), i.p., E12.5 and 1.5 mg/kg poly(I:C), i.p., E17.5	↓ Sociability, spontaneous alternation	10–20 mg/kg suramin, i.p., 2 days before testing	(201)
C57BL/6J mice	3 mg/kg poly(I:C), i.p., E12.5 1.5 mg/kg poly(I:C), i.p., E17.5	↓ Sociability ↑ Marble burying, self-grooming	30 mg/kg JNJ47965567, i.p., (not specified) Genetic removal of *P2rx7*	(202)
C57BL/6J mice	0.25 U/kg poly(I:C), i.p., E12.5 and 0.125 U/kg poly(I:C), i.p., E17.5	↓ Sociability	500 nM clonazepam, i.c., single injection in bilateral ACC	(203)
CD1 mice	5 mg/kg poly(I:C), i.p., E12.5 or E17.5	↓ Sociability, reversal learning	—	(204)
ddY mice	5 mg/kg poly(I:C), i.p., E12–17	↓ Social preference	TPPU, 15 mg/L in drinking water, E12–P21	(205)
ddY mice	5 mg/kg poly(I:C), i.p., E12–17	↓ Sociability, social preference	Maternal diet enriched with glucoraphan, E5–P21	(206)
Sprague-Dawley rats	4 mg/kg poly(I:C), i.v., E15	↓ Sociability	—	(207)
C57BL/6 mice	75 μg/kg LPS, i.p., E11.5–12.5	↓ Sociability (F) ↑ Marble burying (M)	—	(194)
C57BL/6N mice	50 μg/kg LPS, i.p., E14	↓ Sociability, USVs ↑ Marble burying	Maternal injection of anti-IL-17A antibody, i.p., E14	(208)
C57BL/6 mice	75 μg/kg LPS, i.p., E14.5	↓ Sociability, social preference	Maternal injection of inactivated influenza vaccine, i.m., E2.5	(209)
C57BL/6 mice	100 μg/kg LPS, i.p., E15	↓ Sociability, USV duration ↑ Marble burying, self-grooming	—	(210)
Wistar rats	1 mg/kg LPS, s.c., every other day from E7 to delivery	↓ Social interaction, USVs (M) ↑ USVs (F)	—	(211)
Wistar rats	100 μg/kg LPS, i.p., E9.5	↓ Social interaction ↑ Self-grooming	0.8 mg/kg ω-3 polyunsaturated fatty acid, p.o., P30–51	(212)
Wistar rats	100 μg/kg LPS, i.p., E9.5	↑ Self-grooming	—	(213)
Wistar rats	500 μg/kg LPS, i.p., E9.5	↓ Sociability ↑ Marble burying	—	(214)
Sprague-Dawley rats	1.5 mg/kg LPS, i.p., E12	↓ Sociability, social preference	—	(215)
Wistar rats	500 μg/kg LPS, i.p., E16	↓ Social interaction, USVs ↑ Head-dipping	—	(216)
C57BL/6 mice	5 μg IL-6, i.p., E12.5	↓ Sociability	Maternal administration of S31–201, i.p., or diosmin, p.o., E12.5	(217)
C57BL/6J mice	20 μg/kg IL-6, i.p., E12–16	↓ Sociability	—	(218)
C57BL/6J mice	30 μg/kg IL-6, i.p., E12.5–16.5	↓ Sociability, social preference, USVs ↑ Self-grooming	0.1 mg/kg melanotan-II, i.c.v., 7 days	(219)
C57BL/6J mice	0.1 pM pCpG-Mu*il17a*, i.v.	↓ Social preference, social recognition	—	(220)

*1,25OHD, 1α-25 dihydroxycholecalciferol; E, embryonic day; i.c., intracerebral; i.c.v., intracerebroventricular; i.n., intranasal; i.p., intraperitoneal; i.v., intravenous; LA, lateral amygdala; LS, lateral septum; P, postnatal day; p.o., per os; s.c., subcutaneous; USVs, ultrasound vocalizations; M, male; F, female*.

Pathogens that are administered in pregnant rodents range from viruses to bacteria and parasites, and their effects on ASD-related behavioral changes have been examined. Human influenza virus infection in pregnant mice on E9 reduced social interaction (174, 175). Offspring that were exposed to high virus titers exhibited lower 5-HT levels through an increase in metabolism and decrease in levels of oxytocin (175). An injection of group B Streptococcus, a major bacterial pathogen in pregnant women and neonates, impaired social behavior, decreased social interaction, and decreased USVs when inoculated in late gestation in rats (179, 180). These deficits were accompanied by white matter damage in the external capsule and corpus callosum (179, 180). Mice that were born to mothers that were infected with *Mycobacterium tuberculosis* on E12.5 exhibited deficits in approach to social novelty and social preference, and these effects were associated with high plasma IL-6 and IL-17A levels (176). A maternal injection of the soluble tachyzoite antigen of *Toxoplasma gondii* on E14.5 also elicited MIA and produced ASD-like behavior, such as impairments in social approach, an increase in self-grooming, and an increase in marble-burying behavior in offspring (178). Thus, maternal infection in mid- to late-gestation is considered to lead to ASD, regardless of the specific pathogen.

Given that different pathogens cause ASD-like behavioral alterations in rodents, common downstream mechanisms have been sought. Substances that induce MIA include the synthetic double-strand RNA polyinosine-polycytidylic acid (poly(I:C)), which mimics viral infection, lipopolysaccharide (LPS), which mimics bacterial infection, and ILs that are induced by inflammation, such as IL-6. Double-strand RNA is produced by most viruses during replication. A poly(I:C) injection causes the production of proinflammatory cytokines through Toll-like receptor 3 (221). A maternal injection of poly(I:C) on E12.5 promoted an ASD-like phenotype in offspring, including an increase in USVs, a decrease in sociability, and disorganized cortical cytoarchitecture (195). Offspring from LPS-injected mothers on E14 exhibited a decrease in USVs, an increase in marble burying behavior, and a decrease in interest in a novel mouse (208). These findings suggest that MIA models that use agents that mimic MIA contribute to revealing the detailed mechanisms of MIA-related ASD.

Infection or poly(I:C) induces the production of IL-6, which can cross the placenta and affect the fetal brain (222, 223). A maternal injection of IL-6 on E12.5 was sufficient to cause behavioral alterations of preference for social novelty in offspring. The co-administration of anti-IL-6 antibody ameliorated these alterations, but anti-IFN-γ antibody did not. Moreover, maternal poly(I:C) treatment did not disrupt social behavior in IL-6 knockout offspring (186). Parasitic MIA also resulted in deficient social recognition memory that was associated with IL-6 upregulation. IL-6 increased at both the mRNA and protein levels in the brain, and serum IL-6 levels were higher in response to *Toxoplasma gondii* antigen (178). Excessive maternal levels of IL-6 may thus play a role in triggering a cascade of events that lead to ASD in offspring.

Supporting the association between ASD and such cytokines as IL-6 and IL-17, experimental models of MIA-induced ASD have elucidated the role of IL-17 in the development of ASD. A poly(I:C) injection stimulated IL-17A production in dams, followed by higher IL-17A mRNA expression in the fetal brain, an ASD-like phenotype, and cerebral pathological changes in offspring (195). An injection of LPS elevated IL-6 and IL-17A levels only in pregnant mice but exerted ASD-like behaviors in offspring, including a decrease in USVs and lower interest in a novel mouse (208). Similar increases in IL-6 and IL-17A levels were observed in a mouse model of *Mycobacterium tuberculosis* infection, accompanied by behavioral alterations in offspring and increases in the mRNA expression of *NRXN1* and *NLGN1* (176).

Further studies revealed the critical role of fetal exposure to IL-17A but not IL-6 in MIA-associated ASD. The administration of IL-6 in pregnant mice on E12.5 altered social behavior in offspring (186), but an intraventricular injection of IL-6 into the fetal brain on E14.5 did not produce an ASD-like phenotype (195). Instead, an intraventricular injection of IL-17A in the fetal brain on E14.5 caused social deficits and cortical disorganization in wildtype pups (195). The MIA-associated phenotype was blocked by abolishing IL-17A secretion by deletion of the *ROR*γ*t* gene from Th17 cells (195), maternal pretreatment with an anti-IL-17A antibody (208), and deletion of the *IL-17Ra* gene in pups (195). The mechanisms of elevations of IL-17A that lead to ASD may involve alterations of the function of regulatory T (Treg) cells. Mice that were exposed to *Toxoplasma gondii*-induced MIA exhibited higher percentages of Th1 and Th17 cells but a lower percentage of Treg cells. The adoptive cell transfer of Treg cells from MIA mothers at 8 weeks of age largely reversed the abnormal behavioral phenotypes of offspring as early as at 9 weeks (178).

Microglial activation following MIA may be also be involved in ASD development, but few studies have investigated direct effect of alteration of microglial function on ASD-related behavioral deficits in offspring. Morphological changes suggest that microglial activation is not fully consistent in mouse models. Daily injection of IL-6 from E12.5 to delivery resulted in morphological changes of microglia, though not associated with ASD-like behavioral deficits (224). Poly(I:C) administration on E12 and E15 impaired direct social interaction and increased velocity of microglial process motility, but morphological changes was not observed (189). Another study showed that single injection of poly(I:C) effectively produced an ASD-like decrease in sociability and increase in stereotypy, but the superimposition of postnatal hypoxia/ ischemia (HI) insult was needed to alter microglial morphology (225). Microglial infiltration in the brain may be difficult to detect. In two models that produced ASD-like behavioral deficits following MIA [i.e., mice that were exposed to two doses of poly(I:C) on E12.5 and E17.5 (211) and rats that were exposed to daily injections of LPS from E7 to delivery (210)], microglial infiltration in the brain was not detected. In Chen et al., HI superimposition on MIA induced microglial infiltration in the hippocampus (225).

Despite the aforementioned limited histopathological observations of microglia, synapses and neurons that are exposed to MIA exhibit alterations that suggest microglial activation. An increase in spine density that was attributable to a reduction of spine pruning was found in the dentate gyrus in mice given LPS on E15 (226) and in the hippocampus in mice that were given 2 doses of poly(I:C) on E12.5 and E17.5 (211). Subchronic IL-6 exposure from E12.5 to delivery in mice delayed the migration of GABAergic progenitors (224). Parvalbumin-positive neurons in the hippocampus were entrapped in perineuronal nets following MIA alone (225), which may impair synaptic plasticity. A single injection of poly(I:C) on E12 reduced parvalbumin-positive cells and impaired GABAergic signaling in the dentate gyrus, which was associated with social withdrawal and deficient spatial memory (227). In this model, spatial memory and abnormal histology were restored by minocycline, which inhibits microglial activity (227). In the combined MIA/HI mouse model of ASD, the pharmacological inhibition of monocyte infiltration after HI insult prevented ASD-like behaviors (225). Interestingly, MIA-associated behaviors, including ASD-like social impairments, were recovered by exercise in adulthood, which was correlated with the normalization of synapse density and suggested intact microglial function in mice (211). Microglial activation and infiltration in the MIA-exposed brain may link high cytokine levels and the development of ASD, and MIA-induced ASD in offspring could be ameliorated by prenatal or postnatal immunological interventions.

## Hypertensive Disorders of Pregnancy

Hypertensive disorders of pregnancy (HDP) comprise a collection of hypertensions in pregnant women. They are divided into (a) hypertension that arises *de novo* at or after 20 weeks of gestation and (b) hypertension that is known before pregnancy or present in the first 20 weeks of gestation (228). Hypertensive disorders of pregnancy that are accompanied by proteinuria or other maternal organ dysfunction, such as acute kidney injury, are referred to as pre-eclampsia. HDPs occur in ~5–8% of all pregnancies (229). Epidemiological studies reported an association between HDP and ASD. Both maternal chronic hypertension before pregnancy and gestational hypertension are associated with an ~40% increase in the odds of ASD in offspring (23, 230). Pre-eclampsia also increases the risk of ASD (231), but the presence of organ dysfunction in pre-eclampsia does not appear to be an additional risk factor (23, 230). Research on HDP has been performed by generating animal models. The central focus of HDP research has been on maternal pathophysiology and fetal growth failure. In contrast, there are sparse findings on how HDP affects fetal brain development and results in ASD-like social deficits in offspring.

### Reduced Uteroplacental Pressure

Animal models of HDP were first established in rabbits, dogs, and monkeys by constricting the terminal aorta. This method is called reduced uteroplacental pressure (RUPP) (232). Later, this method was applied to rats and mice (233). The procedure was also improved so that uterine arteries were occluded instead of the abdominal aorta, resulting in the avoidance of hindlimb paraplegia in classic RUPP models (234). In addition to main manifestations of HDP, such as maternal hypertension, proteinuria, and fetal growth restriction (232–234), fetal brains also exhibited changes in response to HDP exposure, but their direct relevance to ASD remains unclear. Alteration of the brains of guinea pigs born to RUPP mothers included smaller volumes of the basal ganglia and impairments in prepulse inhibition at 12 weeks (235). Pregnant rats that underwent RUPP also exhibited immunological changes that are similar to MIA, including high levels of IL-6 and IL-17A that are produced by CD4^+^ T cells (236) and an increase in the number of Th17 cells (237). IL-17 recombinant receptor C was used to suppress Th17 cells and resulted in blunted hypertension and the recovery of pup and placenta weight (237). Placental hypoperfusion in animal models of RUPP-induced HDP may mediate morphological and functional changes that are related to MIA-induced ASD in offspring.

### Angiotensin II

Numerous clinical observations have indicated a relationship between HDP and angiotensin (AT). Patients with HDP exhibited greater vascular reactivity to AT II (238), and serum from pregnant patients with HDP but not those with essential hypertension contained AT1 autoantibodies (AAs) that could stimulate AT II receptor 1 (239). AT1-autoantibodies that were isolated from women with HDP reproduce key features of HDP in mice (240), and their pharmacological blockade normalized maternal blood pressure during pregnancy (241). Exposure to LPS during pregnancy gave rise to features that are relevant to HDP in rats offspring, which was prevented by treatment with the AT II receptor inhibitor losartan (242). Infusions of AT II in pregnant mice stimulated the production of soluble fms-like tyrosine kinase-1, a circulating antagonist of vascular endothelial growth factor and placental growth factor (243), and IL-6 in the placenta (244). In summary, AT II infusion is sufficient to induce HDP and IL-6 production, and AT II inhibition is sufficient to prevent LPS-induced HDP, suggesting that the stimulation of AT II signaling underlies MIA and HDP. Thus, excessive AT II activity in HDP might give rise to ASD through an increase in cytokine production as observed in MIA. Further research is required to elucidate the neurological phenotype that is induced by AT II in these models.

### Vasopressin

In contrast to the AT-related HDP model, pregnant women with preeclampsia are reported to exhibit lower activity of the renin-angiotensin system (245). Women with HDP exhibited higher levels of copeptin, a pro-segment of arginine vasopressin (AVP) (246), raising the hypothesis that high AVP levels during pregnancy play a role in the pathogenesis of HDP. Infusions of AVP during pregnancy resulted in an HDP phenotype in mice (247). The simultaneous inhibition of AVP receptors 1 and 2 abolished the AVP-induced elevation of blood pressure, whereas blocking each receptor alone resulted in the insufficient normalization of blood pressure in mid- and late-pregnancy (247). Infusions of AVP during pregnancy increased IL-17 levels in maternal plasma and the placenta (248), suggesting that IL-17 may also affect fetal brain development following AVP loading. To date, however, only one study has reported neurological and behavioral consequence of AVP-infused HDP in offspring (249). Both male and female offspring that were exposed to AVP on P7 had a smaller neocortex, but caudate-putamen volume decreased in males only and increased relative to the neocortex in females. At the behavioral level, male offspring that were exposed to AVP exhibited an increase in anxiety-like behavior in the elevated plus maze test, but females did not. Social behavior was analyzed in males only, in which an increase in sociability was observed in AVP-exposed males compared with controls (249). Further research on ASD-like phenotypes in the AVP infusion model of HDP is necessary to determine whether AVP-induced HDP recapitulates the higher risk of ASD in humans.

### Serotonin Metabolism in the Placenta

Women with HDP have high 5-HT levels in blood (250) and the placenta (251). Such a hyperserotonemic state is attributable to a reduction of activity of monoamine oxidase A and not a defect in 5-HT transport in the placenta (251, 252). Serotonin levels correlated with the severity of maternal hypertension (251), suggesting a causal role for hyperserotonemia in HDP. Serotonin exerts a contraction effect on placental vascular smooth muscle, which may be a primary way 5-HT plays a role in HDP (253, 254). Chorionic arteries from HDP women exhibited a lower contraction response to the *in vitro* perfusion of 5-HT (255), presumably because of chronic exposure to excessive 5-HT during pregnancy. Ketanserin, a 5-HT_2A_ receptor antagonist, attenuated arterial contraction that was stimulated by 5-HT in both normal and HDP samples (255). Moreover, 5-HT administration in normal pregnant rats produced HDP-like alterations, such as a reduction of growth of the placenta and fetus (256). Continuous hyperserotonemia in *Sert*^−/−^ mice, which was 2-fold higher than controls, produced smaller placentas, marked cell death, and necrotic lesions that were similar to placentas from HDP women (257). Undetectable 5-HT levels in *Tph*1^−/−^ mice also resulted in smaller placentas and cell death, although these effects were much milder than in *Sert*^−/−^ mice (257). These similar and contrasting findings from hyperserotonemic and hyposerotonemic models suggest a more deleterious effect of hyperserotonemia on the placenta and possibly on the fetus during pregnancy. As discussed in Section SSRIs above, the relationship between high 5-HT levels and ASD has been demonstrated in different animal models. Investigations of these models in view of HDP may improve our understanding of how HDP causes ASD in offspring.

## Advanced Parental Age

Advanced parental age does not appear to accompany the visible effects that are discussed above, such as the effects of medication use and the inflammatory response during pregnancy. A detailed population study sought to identify prenatal and perinatal risks of ASD and found higher parental ages of ASD children compared with non-ASD children (258), prompting further studies of the association between parental age and the risk of ASD.

### Effects of Paternal and Maternal Age and Their Interaction on ASD

The risk of higher paternal age for ASD have been consistently reported. In a study in Israel, the odds ratio (OR) of ASD in offspring marginally increased for paternal ages of 30–39 years and significantly increased (OR = 5.75) for paternal ages of 40 years and older when adjusted for maternal age and compared with paternal ages of <30 years (259). A cohort study in Sweden also reported an association between paternal age and the risk of ASD, which began to increase at the paternal age of 30 years (260). The authors then confirmed this association in a meta-analysis of Western and non-Western cohorts, which revealed a dosage effect on the OR of ASD in offspring (1.22 for paternal age 30–39 years, 1.58 for paternal age 40–49 years, and 2.66 for paternal age 50 years and older) (260). A similar linear increase in the risk of ASD according to paternal age was also found in a large-scale cohort study (261). A recent meta-analysis showed that high paternal age was associated with a 55% higher risk of ASD (262).

Maternal age also appears to affect the risk of ASD in offspring, though in a different manner compared with paternal age. In a population study, logistic regression analysis was conducted that included maternal but not paternal age as regression coefficients. This study found that having children with ASD was related to higher maternal age (258). However, the effect of advanced maternal age was marginal when adjusted for parental age (259). Later studies found that the risk of ASD in offspring increased in mothers who were 30 of age or older (261) and 35 years of age or older (263). When the ages of both mothers and fathers were adjusted, the risk of ASD in offspring began to increase in mothers who were 30–39 years old and evident in mothers who were 40 years old (264).

In contrast to consistent findings that advanced parental age elevates the risk of ASD in offspring, remaining to be clarified is whether lower parental age heightens the risk of ASD in children. A cohort study in Sweden found that the risk of ASD for younger mothers was comparable to a reference group (29 years old), whereas children of younger fathers had a lower risk of ASD (261). Another cohort study of European and non-European countries reported a higher risk of ASD in children of mothers who were younger than 20 years old, whereas no association was observed for younger fathers (264). According to a meta-analysis from North America, Europe, Asia, and Oceania, the lowest paternal and maternal age categories were associated with a lower risk of ASD (262). Lower parental age might have a protective effect against the risk of ASD in offspring, which should be investigated further.

Remaining to be determined are which maternal and paternal ages have the greatest impact on the risk of ASD. A Swedish population study reported a higher OR of ASD, particularly ASD with intellectual disability, that was associated with advanced maternal age (2.04 for mothers aged 40–45 years and 1.18 for fathers aged 40–44 years) (261). Another study reported a higher risk of ASD for fathers aged 40–49 years (OR = 1.52) compared with mothers of the same age group (OR = 1.15) (264). A recent meta-analysis found a comparable risk of ASD for the highest age group (OR = 1.41 for mothers and 1.55 for fathers) (262). Further research is needed to distinguish the effects of maternal and paternal ages.

In summary, both advanced maternal and paternal age are likely related to a higher risk of ASD. Data have been insufficient or inconsistent with regard to differences in the impact of maternal and paternal ages and their interaction on ASD.

### Modeling Advanced Parental Age in Rodents

The influence of advanced parental age has been investigated in rodent models, mainly for older fathers. Paternal age did not affect the general health of pups, such as the number of pups, body weight, and early mortality (265). Mice from older fathers exhibited alterations of behavioral phenotypes. These offspring took longer to attain the righting reflex, exhibited a decrease in spontaneous motor activity, deficient memory retention in the passive avoidance test (265), and decreases in ambulatory distance and prepulse inhibition (266). ASD-related behavior was also found in advanced paternal age models. Male mice from older fathers exhibited impairments in social preference (267), the deficient discrimination of social novelty (267, 268), an increase in self-grooming (268), and increases in or alterations of USVs that are suggestive of ASD (268, 269). The magnitude of social deficits correlated with paternal age. Lower social interaction was observed in offspring of 40-week-old fathers and further decreased in offspring of 48-week-old fathers (270). Autism spectrum disorder-like social deficits that are caused by advanced paternal age may transmit to the second generation. When both parents were born to fathers that were 12 months of age or older, the offspring exhibited a decrease in sociability, an increase in repetitive behavior, and anxiety-like behavior (267, 268).

Little is understood about what accompanies behavioral changes that are observed in offspring of older fathers. Advanced paternal age may produce morphological changes in the brain in offspring, but the findings are inconsistent. Male mouse offspring of 12- to 18-month-old fathers had a higher rostral cortical volume and lower lateral ventricle volume, but social interaction was unaffected (271). A recent study that used male mice that were older than 12 months of age found that their offspring emitted abnormal USVs. This finding was associated with a smaller cortical thickness of layer 6 of the primary motor cortex, suggesting that alterations in the motor cortex impair vocal communication (269). A possible mechanism of paternal age-induced ASD may be related to alterations of sperm DNA methylation. Older fathers and their offspring shared hypomethylation in regions that flank CpG island promotors, which was not found in younger fathers and their offspring (266). Whole-genome DNA methylome analyses of sperm identified hypomethylated genomic regions that were enriched in RE1-silencing transcription factor/neuron-restrictive silencer factor binding motifs (265). The treatment of young male mice with the DNA-demethylating drug T5-Aza reproduced DNA hypomethylation in sperm, and their offspring exhibited alterations of USV patterns (269). Alterations of DNA methylation patterns in sperm may contribute to the effect of advanced paternal age on ASD in offspring.

Analyzing advanced maternal age in animal models is challenging because aged females may exhibit changes in nursing behavior. For example, 30-week-old female mice exhibited enhanced nest-building performance during pregnancy and lactation (272). Cesarean section and cross-fostering were applied to avoid confounding effects of alterations of nursing behavior in 15- to 18-month-old female mice, but their pups still exhibited more USVs on P8 and an increase in anxiety-related behavior in the elevated plus maze (273). Associated with the behavioral changes, pups that were born to aged female mice exhibited high hippocampal mRNA expression of genes that are related to ASD, such as *Ada* (which influences ASD development) and *Egr2* (which influences ASD severity). Several other genes were enriched in the Gene Ontology analysis, including “protein folding” and “protein post-translational modification” (273). Younger maternal age in mice (32–35 weeks) also affected cognitive functions in offspring, including impairments in learning in the passive avoidance test, spatial memory in the Morris water maze test, and memory in the novel object recognition test (274). Interestingly, a marked decrease in expression of the vitamin D receptor gene was found in the placenta in aged female mice and their offspring (274). When these aged female mice received vitamin D supplementation before pregnancy, their offspring exhibited intact learning and memory (275). Advanced maternal age likely affects cognitive function in offspring, and possible ASD-like social deficits in these models should also be investigated.

## Concluding Remarks

We discussed the various contributions of environmental risk factors to the development of ASD. Prenatal exposure to VPA and MIA have been extensively investigated in epidemiological and biological studies. The findings indicate the critical role of these risk factors in producing ASD. Hypertensive disorders of pregnancy and advanced maternal age elevate the risk of ASD, but remaining unclear is how these risk factors give rise to ASD-like cognitive dysfunction. The association between maternal SSRI use and ASD in offspring is inconclusive, but rodent models that show alterations of 5-HT metabolism provide a plausible rationale for this association. Future research is expected to develop therapeutic interventions for ASD that target environmental risk factors.

## Author Contributions

AS wrote the manuscript. HK-M, MT, YK, and KI critically reviewed the manuscript. All authors contributed to the article and approved the submitted version.

## Funding

This work was supported by a grant from the Japan Society for the Promotion of Science (JSPS) KAKENHI (no. 21H03028).

## Conflict of Interest

The authors declare that the research was conducted in the absence of any commercial or financial relationships that could be construed as a potential conflict of interest. The reviewer RK declared a shared affiliation with two of the authors, MT and YK, to the handling editor at the time of review.

## Publisher's Note

All claims expressed in this article are solely those of the authors and do not necessarily represent those of their affiliated organizations, or those of the publisher, the editors and the reviewers. Any product that may be evaluated in this article, or claim that may be made by its manufacturer, is not guaranteed or endorsed by the publisher.
